# Altered static and dynamic functional network connectivity in primary angle-closure glaucoma patients

**DOI:** 10.1038/s41598-024-62635-6

**Published:** 2024-05-22

**Authors:** Yuanyuan Wang, Yongqiang Shu, Guoqian Cai, Yu Guo, Junwei Gao, Ye Chen, Lianjiang Lv, Xianjun Zeng

**Affiliations:** 1 Department of Radiology, The First Affiliated Hospital of Nanchang University, Nanchang, China; 2 Positron Emission Tomography (PET) Center, The First Affiliated Hospital of Nanchang University, Nanchang, China

**Keywords:** Primary angle-closure glaucoma, Magnetic resonance imaging, Static functional network connectivity, Dynamic functional network connectivity, Brain functional network, Medical research, Biomarkers

## Abstract

To explore altered patterns of static and dynamic functional brain network connectivity (sFNC and dFNC) in Primary angle-closure glaucoma (PACG) patients. Clinically confirmed 34 PACG patients and 33 age- and gender-matched healthy controls (HCs) underwent evaluation using T1 anatomical and functional MRI on a 3 T scanner. Independent component analysis, sliding window, and the K-means clustering method were employed to investigate the functional network connectivity (FNC) and temporal metrics based on eight resting-state networks. Differences in FNC and temporal metrics were identified and subsequently correlated with clinical variables. For sFNC, compared with HCs, PACG patients showed three decreased interactions, including SMN-AN, SMN-VN and VN-AN pairs. For dFNC, we derived four highly structured states of FC that occurred repeatedly between individual scans and subjects, and the results are highly congruent with sFNC. In addition, PACG patients had a decreased fraction of time in state 3 and negatively correlated with IOP (p < 0.05). PACG patients exhibit abnormalities in both sFNC and dFNC. The high degree of overlap between static and dynamic results suggests the stability of functional connectivity networks in PACG patients, which provide a new perspective to understand the neuropathological mechanisms of optic nerve damage in PACG patients.

## Introduction

Glaucoma comprises a group of progressive optic degenerative diseases marked by visual field loss and optic nerve atrophy, emerging as a prominent cause of irreversible blindness globally^1^. Primary glaucoma categorizes into primary angle-closure glaucoma (PACG) and primary open-angle glaucoma (POAG), contingent on angle closure. PACG is more prevalent in Asian populations, exhibiting a heightened incidence of blindness and a more substantial societal burden compared to POAG^2^. Despite this, the pathophysiological mechanisms of PACG remain unclear. Elevated intraocular pressure (IOP) is the primary risk factor for glaucoma, it’s damage to retinal ganglion cells (RGCs) playing a key role in glaucoma's development^3,4^. Notably, RGCs, developmentally and anatomically extensions of the central nervous system, have attracted attention. Many neuropathological studies have revealed that glaucoma is a trans-synaptic neurodegenerative disease involving the brain, indicating that it should be classified not only as an ophthalmological condition but also as an encephalopathy^5–8^.

Recently, there has been a growing utilization of resting-state functional magnetic resonance imaging (rs-fMRI) to scrutinize the intrinsic functional activity in individuals affected by glaucoma. Resting-state functional connectivity (RS-FC), an analytical approach investigating statistical correlations among signals emanating from either entire or localized brain regions, demonstrates significant promise in elucidating synchronous brain activity. Wang et al.^9^ found extensive abnormal rs-FC between the thalamus and visual and extravisual brain areas. Tong et al.^10^ demonstrated that there is disturbed interhemispheric resting-state functional connectivity in the vision-related brain areas of individuals with PACG. Besides, our research team previously observed that patients with PACG exhibited brain dysfunction in various areas, and displayed distinct spatial variations in both proximal and distal functional connection density (FCD)^11^. In addition to regional changes in brain function, the characteristics of brain networks in PACG patients also changed. Cai et al.^12^ found that the degree centrality (DC) values of bilateral visual cortex and left anterior central gyrus in PACG patients after surgery were significantly higher than those before surgery. Chen et al.^13^ found that PACG patients exhibited extensive dysfunction in the visual network, auditory network, default mode network, and cerebellar network, which may provide new clues to the neural mechanisms of optic atrophy in PACG patients. The above studies suggest that patients with PACG exhibit specific functional irregularities within interregional and global brain networks. However, the alterations in functional network connectivity (FNC) in PACG patients remain poorly understood.

Static FNC (sFNC) is utilized to assess temporal correlations among brain regions; however, this analysis overlooks the variability in neurophysiological activities that individuals may exhibit across different situations and times^14^. Moreover, emerging evidence underscores the intricate nature of the brain as a complex system with dynamic properties and temporal dependencies^15–17^. Changes in dynamic FNC (dFNC) are linked to specific psychiatric conditions, cognitive states, and neurological diseases^18–20^. Integrating sFNC and dFNC into a unified analysis is crucial, as the former can be conceptualized as an aggregation of ongoing dynamic changes. Wang et al.^21^ illustrated sFNC alterations between default mode network (DMN) and visual network (VN) in POAG patients. However, the modifications in both sFNC and dFNC remain largely unexplored in patients with PACG.

The present study aimed to explore a potential neuromechanism of PACG patients by using a combination of resting-state data and ICA approach. We hypothesized that PACG patients demonstrate aberrant sFNC and dFNC patterns and these sFNC and dFNC aberrances correlate with clinical ophthalmic parameters.

## Materials and methods

### Participants

This study employed an observational case-control design, with sample size calculation based on a two-sample approach to test differences between patients with Primary angle-closure glaucoma (PACG) and healthy controls (HCs). The power calculation was conducted using the G-Power3.1 software (http://www.gpower.hhu.de/). Given the incidence of PACG patients, we opted for a larger effect size to enhance the likelihood of detecting significant differences with a smaller sample size. Specifically, we set the parameters as follows: Tails = two; Effect size d = 0.7; αerror probability = 0.05; Power (1-βerror probability) = 0.8; Allocation ratio N2/N1 = 1. Consequently, the calculated sample size for both groups is 34.

A total of 40 right-handed PACG patients were recruited from the Ophthalmology Department of the First Affiliated Hospital of Nanchang University, following specific criteria aligned with the European glaucoma guidelines^22^. Inclusion criteria encompassed: (a) monocular or bilateral angle-closure confirmed by gonioscopy; (b) glaucoma-associated visual field defects, such as tubular vision and nasal hemianopia; (c) optic nerve cup-to-disc ratio > 0.6, determined through funduscopic examination; and (d) no history of medical or surgical glaucoma treatment. Exclusion criteria included: (a) diagnosis of other glaucoma types or ocular diseases; (b) history of acute seizures; (c) underlying diseases like hypertension or diabetes; (d) prior surgical glaucoma treatment; (e) incomplete MRI scan or clinical assessment data; and (f) contraindications for MRI, such as metal implants or claustrophobia. Ultimately, 34 PACG patients (16 males and 18 females) were included in the study, undergoing comprehensive ophthalmic examinations, including goniometric and tonometric intraocular pressure (IOP) measurement, fundus optical coherence tomography, and visual field testing. We recruited and selected 33 healthy subjects to serve as healthy controls (HCs; 16 males and 17 females). Inclusion criteria were as follows: (a) no typical glaucoma symptoms; (b) no ocular disease or other systemic disease; (c) age and gender matched with PACG; and (d) right-handed.

This study adhered to the principles outlined in the Declaration of Helsinki, and approval for the study protocol was granted by the Human Research Ethics Committee of the First Affiliated Hospital of Nanchang University (YLP20231050). Prior to participation, each individual provided written informed consent.

### Data acquisition

Resting-state functional magnetic resonance imaging (rs-fMRI) data were acquired utilizing a 3 T MR scanner (Siemens, Erlangen, Germany) equipped with an 8-channel phased-array head coil at the First Affiliated Hospital of Nanchang University, located in Nanchang city, Jiangxi Province, China. Participants were instructed to remain quietly lying with closed eyes, avoiding sleep, specific thoughts, and any head movement during the scanning session. The acquisition of functional images spanned 8 min, encompassing 240 resting-state volumes, with the following parameters: repetition time (TR) = 2000 ms; echo time (TE) = 40 ms; flip angle = 90°; field of view (FOV) = 240 mm × 240 mm; matrix = 64 × 64; and slice thickness = 4 mm with a 1 mm gap. Structural 3D T1-weighted images were obtained using the three-dimensional turbo fast-echo (3D-TFE) T1WI sequence, featuring the following specifications: TR = 1900 ms; TE = 2.26 ms; flip angle = 9°; FOV = 240 mm × 240 mm; matrix = 256 × 256; number of sagittal slices = 176; and slice thickness = 1 mm.

The MRI data collection for this project is conducted at the Clinical Research Center for Medical Imaging in Jiangxi Province (No.20223BCG74001).

### Data pre-processing

Resting-state fMRI data preprocessing was conducted using the GRETNA software (http://www.nitrc.org/projects/gretna) within MATLAB (version R2018b, MathWorks, Inc., Natick, MA, USA)^23^. The preprocessing procedure comprised the following steps: (1) discarding the initial ten scans to allow for magnetization equilibration, (2) implementing slice timing correction, (3) realigning resting-state data to the first volume to rectify interscan head motions, (4) spatial normalization, involving the registration of functional data to the corresponding structural T1-weighted image and normalization into the standard Montreal Neurological Institute template using nonlinear transformations^24^, and (5) applying smoothing with an isotropic Gaussian kernel of 8 mm full width at half maximum to enhance the signal-to-noise ratio.

### Group ICA

**Figure 1:**
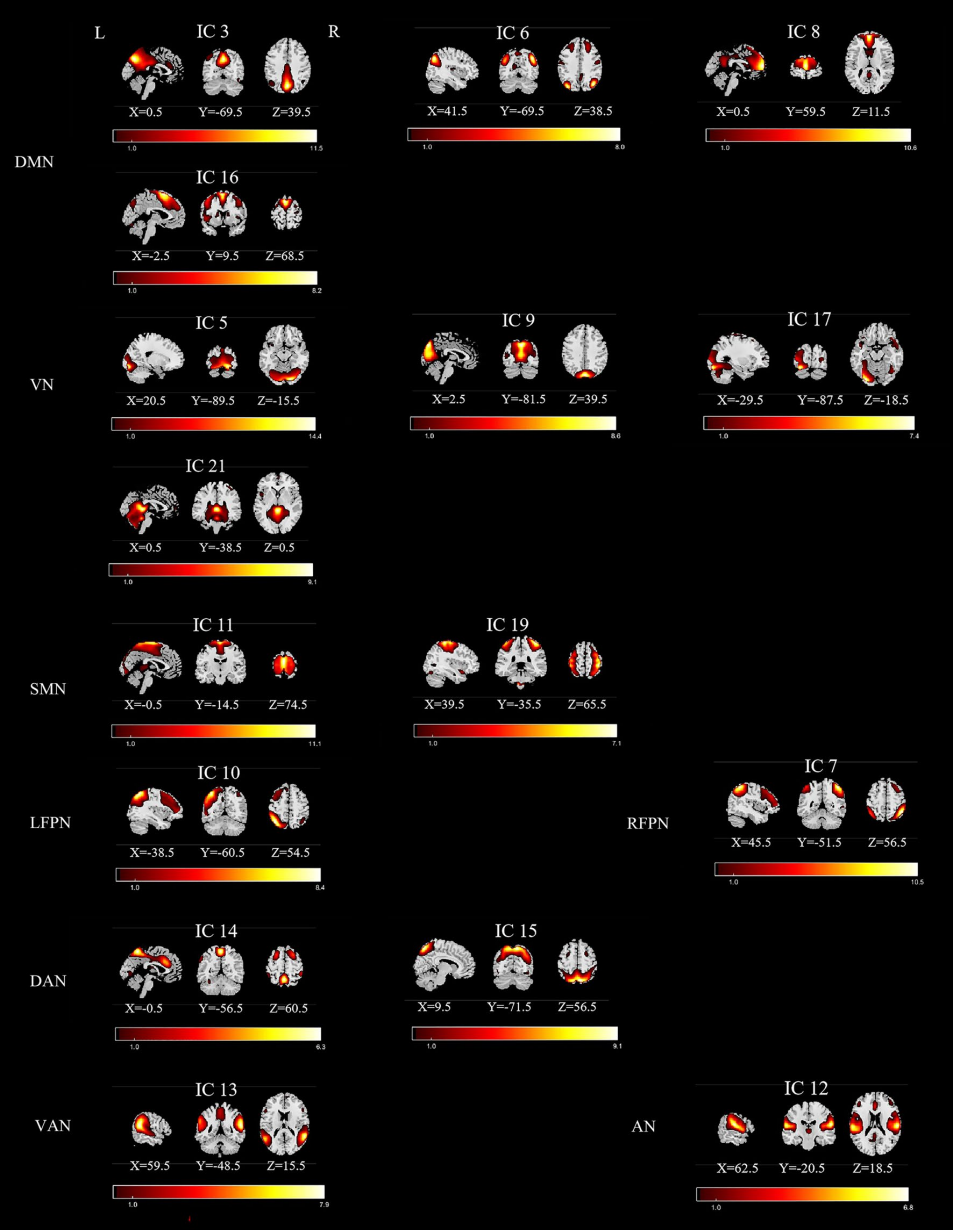
Spatial maps (displayed at the three most informative slices) of 16 independent components (ICs) that have chosen as our networks of interest. R represent right and L represents left. *DMN* default mode network, *VN* visual network, *SMN* sensorimotor network, *LFPN* left frontoparietal network, *RFPN* right frontoparietal network, *DAN* dorsal attention network, *VAN* ventral attention network, *AN* auditory network.

Following data preprocessing, we employed the spatial independent component analysis (ICA) method facilitated by GIFT software (http://icatb.sourceforge.net/)^25,26^ to analyze the data of all subjects. The dataset underwent decomposition, revealing functional networks characterized by unique temporal dynamics. The ICA process encompassed key stages: (1) Component Estimation: The number of independent components (ICs) was determined using the minimum description length (MDL) criteria^27^. (2) Data Downscaling: Principal component analysis (PCA) compressed and downscaled the data. These downscaled data were then subjected to IC decomposition using the infomax algorithm. This iterative process was repeated 20 times in ICASSO (http://research.ics.tkk.fi/ica/icasso/) to ensure robust estimation. (3) Inverse Reconstruction: Subject-specific spatial maps and time courses were obtained through the GICA back reconstruction approach. Intensity values corresponding to each voxel underwent Fisher-z transformation, resulting in data that approximately followed a normal distribution^28^. (4) Component Selection: The Display GUI module in the GIFT software package visualized the obtained components, and ICs associated with cerebrospinal fluid, motor, or vascular evoked pseudoactivation were excluded. Finally, from the 21 components yielded by ICA, 16 were selected for focused analysis in subsequent examinations. We assigned these 16 ICs to eight networks based on a visual examination of the spatial maximum overlap and the stanford functional risk standard template. We then assembled the peak slices of each IC to produce Fig. 1.

### sFNC analysis

**Figure 2:**
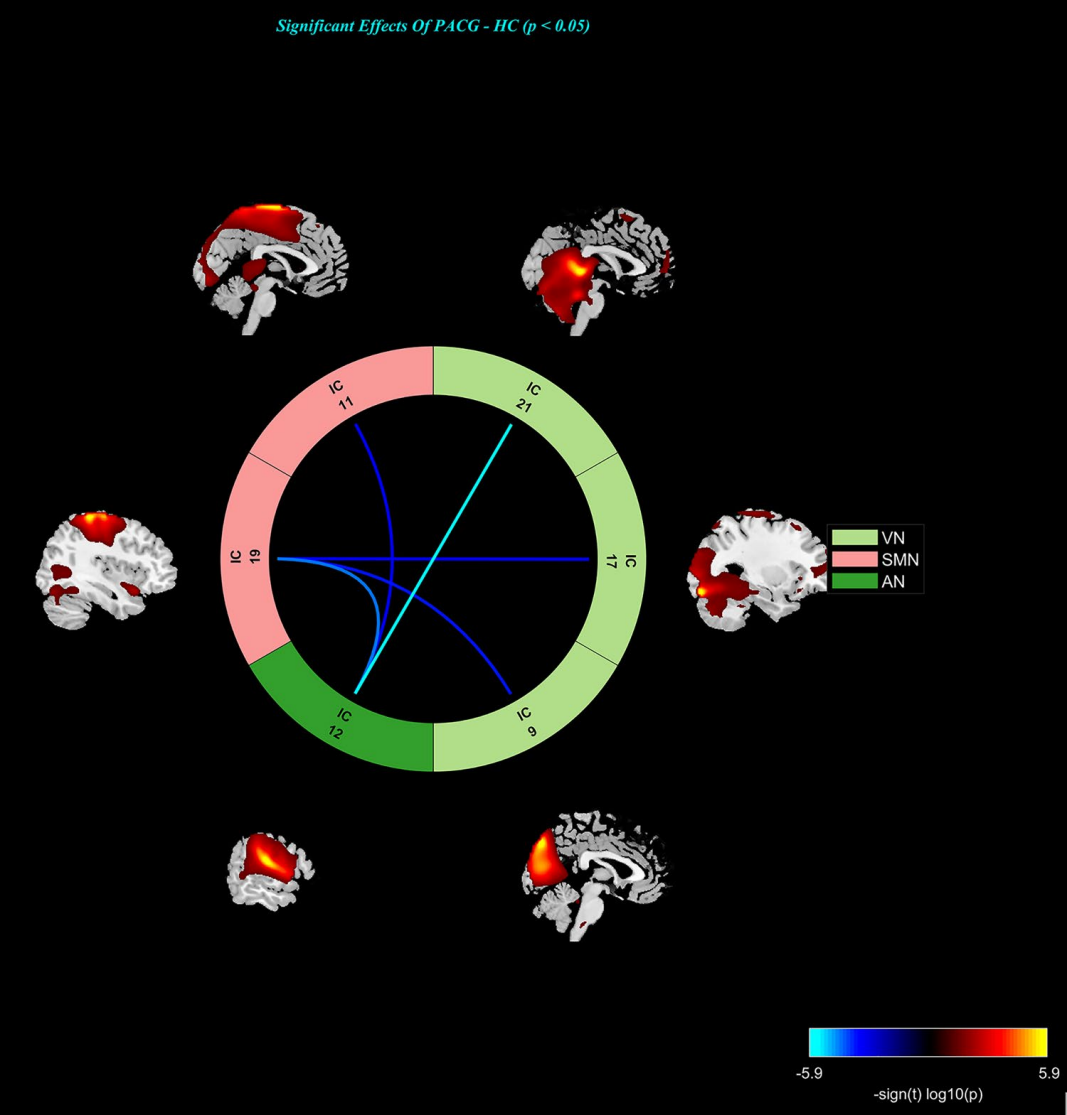
Significant differences in the network connectivity in the auditory network (AN), sensorimotor network (SMN) and visual network (VN) between PACG group and HC group.

The analysis of static functional network connectivity (sFNC) utilized the mancovan toolbox within the GIFT software. Initially, detrending involved addressing linear, quadratic, and cubic trends, alongside despiking to identify outliers. Additionally, a low-pass filter with a cut-off frequency of 0.15 Hz was applied. Subsequently, pair correlations among these independent components (ICs) were computed and subjected to transformation using Fisher’s Z-transform. Within the framework of the general linear model, an estimation of group differences in static FNC was conducted for each pair of resting-state networks, with control variables for age, gender, and educational level. Following adjustments for multiple comparisons using the false discovery rate (FDR), the significance threshold was set at p < 0.05. Finally, the display component in mancovan_toolbox was used to display the sFNC results (Fig. 2).

### dFNC analysis

**Figure 3:**
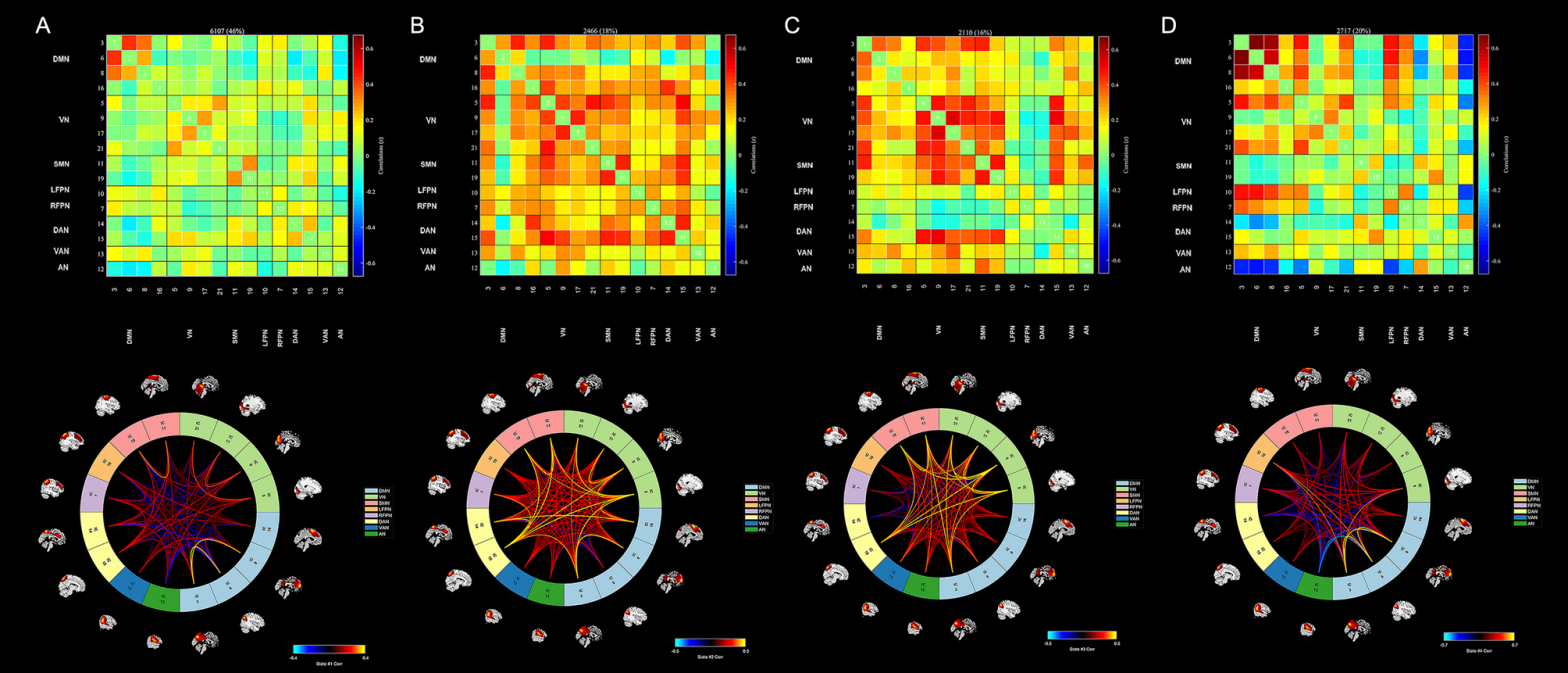
Results of the clustering analysis per state. (**A**–**C**), and (**D**) represent the cluster centers and dFNC diagrams of states 1–4, respectively. The total number of occurrences and percentage of total occurrences are listed above each cluster median.

**Figure 4:**
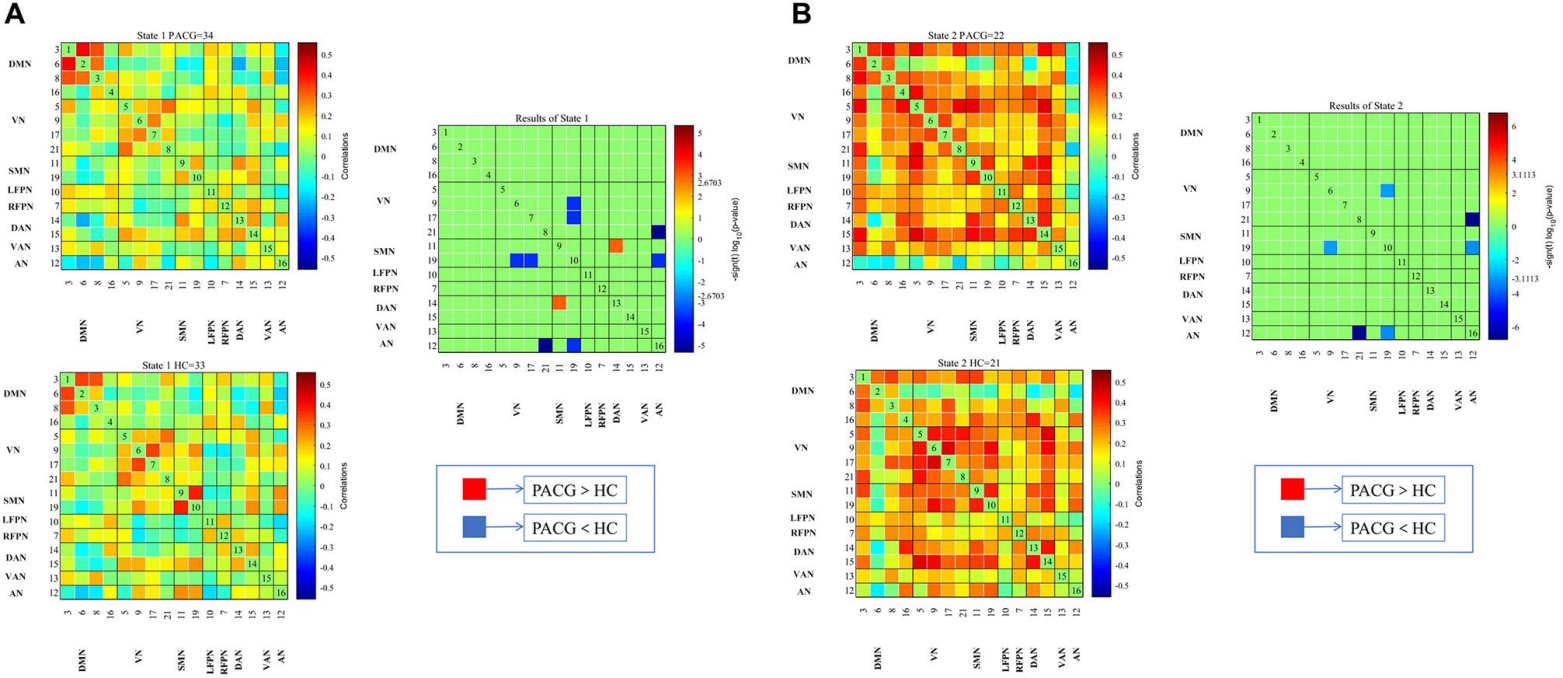
Differences in dynamic functional network connectivity (dFNC) between PACG group and HC group. dFNC assumes that whole brain connectivity sequentially iterates through a finite set of connectivity patterns known as dFNC states. Here only State 1(**A**) and state 2 (**B**) are shown because significant differences between groups (PACG and HC) in dFNC were observed only in these states.

To elucidate the dynamic nature of FNC, we employed the temporal dFNC toolkit within the GIFT software. The computation of dFNC between ICA time courses followed a sliding time-window methodology. Prior research has indicated that effective capture of dynamic state changes in the brain occurs with a window width ranging between 30 and 60 s^29,30^. In our study, the dFNC sliding window size was standardized at 30 TRs (60 s), utilizing a rectangular window convolved with a Gaussian function (= 3 TRs). Subsequently, the dFNC matrices of all subjects underwent clustering through the k-means algorithm, facilitating an evaluation of the frequency and structure of recurring dFNC patterns. The cityblock distance was employed in this analysis to quantify the similarity between different time windows. To enhance the likelihood of the clustering algorithm avoiding local minima, we stipulated a maximum of 500 iterations and 150 repetitions. The identified patterns were classified into four FNC states using the elbow criterion^31^. The dFNC matrix at the centre of each cluster was called the cluster centroid, which can be showed by the display component of the dfnc_toolbox (Fig. 3). Several temporal characteristics were computed, including: (1) the fraction of time, denoting the percentage of each state within the four states, (2) mean dwell time, representing the average time a subject spent in a specific state, and (3) transition number, indicating the frequency with which a subject transitioned between states. The stats component in the dfnc_toolbox can calculate and display the distribution of subjects across the four states, as well as the dFNC values within each state (Fig. 4).

### Statistical analysis

The statistical analysis utilized the SPSS 26.0 software package(https://www.ibm.com/support/pages/downloading-ibm-spss-statistics-26.) (SPSS, Inc., Chicago, IL, USA). Continuous data were presented differently based on their normal distribution. For data conforming to normal distribution, descriptive statistics are expressed as the mean ± SD, and an independent sample t-test was employed. In cases where the data did not conform to normal distribution, the presentation involved the median (quartile), and the analysis employed the Mann–Whitney test. Categorical variables were conveyed as counts, and statistical significance was determined using either the χ^2^-test or Fisher’s test.

The investigation of differences in dFNC between groups employed two-sample *t*-tests for each state, with results subject to correction for multiple comparisons using the false discovery rate (FDR) (p < 0.05). Temporal properties of dFNC states were examined by calculating the fraction of time, mean dwell time in each state, and the number of transitions between states. Additionally, direct analysis of disparities in sFNC and dFNC between the PACG and HC groups utilized the Stats module in the GIFT software package. Two independent samples *t*-tests were employed with FDR correction, and significance was set at p < 0.05, indicating statistically significant differences.

Pearson’s correlation analysis assessed the relationship between altered network attributes (network metrics and temporal attributes) and clinical variables such as IOP, RNFLT, and VA, while controlling for age and gender. Additionally, Spearman correlation analysis examined the association between sFNC values and disease duration among networks. Statistical analysis was conducted using SPSS 26.0, setting the threshold at p < 0.05.

### Ethics approval and consent to participate

The ethics board of the First Affiliated Hospital of Nanchang University approved the study. All participants provided informed consent.

## Results

### Demographics and clinical characteristics

The demographic and clinical ophthalmic characteristics of both PACG group and HC group were shown in Table 1. There were no differences between groups in age, gender and educational level (p > 0.05). While there were significantly differences between groups in IOP, VA, RNFLT, A-C/D and V-C/D (p < 0.001).

**Table 1:** Demographics and clinical characteristics of all subjects.

Condition	PACG(n = 34)	HC(n = 33)	p value
Age(years)	53.12 ± 11.75	52.85 ± 10.61	0.922
Gender(male/female)	16/18	16/17	0.907
Education level(years)	9.44 ± 2.76	9.91 ± 2.7	0.485
Disease duration(years)	0.49(0.08, 2.25)	–	–
IOP(mmHg)	27.34 ± 9.24	15.49 ± 2.01	< 0.001
RNFLT(μm)	86.77 ± 24.96	117.27 ± 8.95	< 0.001
Mean VA	0.53 ± 0.31	1.10 ± 0.21	< 0.001
A-C/D	0.67 ± 0.15	0.46 ± 0.11	< 0.001
V-C/D	0.64 ± 0.18	0.49 ± 0.08	< 0.001

### Networks of interests

From a total of 21 components, 16 Independent components (IC) were chosen as our networks of interest (Fig. 1), which were grouped into the following eight networks: default mode network (DMN) (ICs3,6,8,16), visual network (VN) (ICs5,9,17,21), sensorimotor network(SMN)(ICs11,19), left frontoparietal network (LFPN) (IC10), right frontoparietal network (RFPN)(IC7), dorsal attention network (DAN) (ICs14,15), ventral attention network (VAN) (IC13), auditory network (AN)(IC12).

### Cluster and temporal metrics analysis

**Figure 5:**
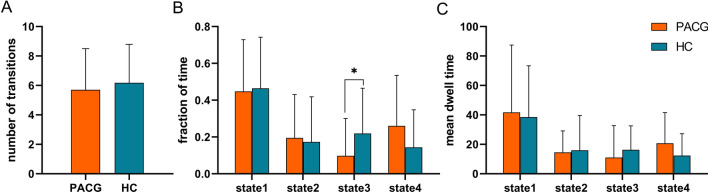
Between-group comparison of dFNC temporal metrics. Bar charts show (**A**) the number of transitions, (**B**) the fraction of time and (**C**) the mean dwell time. *above the bars indicates a significant difference between the two groups, p < 0.05.

Using the k-means clustering method, we derived four highly structured states of FC that occurred repeatedly between individual scans and subjects. Figure 3 shows the four common functional connection states and corresponding cluster centers. The total percentages of these four states in all subjects were different: State 1(46%) accounted for the highest proportion of all time windows and had sparse connectivity; State 2 (18%) showed strong connectivity, exhibiting strong positive connectivity amongst large-scale functional networks; State 3 (16%) had the smallest proportion of all time windows, showing partial connectivity; and State 4 (20%) was characterised by modular connectivity, primarily observed between the DMN, VN and AN. As shown in Table 2 and Fig. 5, the fraction of time in state 3 was significantly shorter in PACG group compared to HC group. However, we did not find any significant group differences in the mean dwell time and number of transitions in each state (p > 0.05).

**Table 2:** dFNC temporal metrics between patients with PACG and healthy controls.

Metrics	PACG(n = 34)	HC(n = 33)	T value	P value
Number of transitions	5.71 ± 2.80	6.18 ± 2.62	− 0.718	0.475
State1				
Fraction of time	0.45 ± 0.28	0.46 ± 0.28	− 0.245	0.807
Mean dwell time	41.73 ± 45.77	38.57 ± 34.78	0.317	0.752
State2				
Fraction of time	0.19 ± 0.24	0.17 ± 0.25	0.369	0.713
Mean dwell time	14.56 ± 14.56	15.92 ± 23.68	− 0.284	0.778
State3				
Fraction of time	0.10 ± 0.20	0.22 ± 0.25	− 2.21	0.031
Mean dwell time	11.00 ± 21.70	16.26 ± 16.27	− 1.119	0.267
State4				
Fraction of time	0.26 ± 0.28	0.14 ± 0.20	1.964	0.054
Mean dwell time	20.69 ± 20.81	12.39 ± 14.84	1.876	0.065

### Aberrance in sFNC and dFNC

For the sFNC analysis, relative to the HC group, the PACG group exhibited significantly decreased interactions in three static connections, including the SMN-AN, SMN-VN and VN-AN connection (Fig. 2).

For the dFNC analysis, significant differences between groups were observed only in state 1 and states 2 (Fig. 4). In state 1, the connectivity between SMN-VN, SMN-AN, VN-AN is decreased, while the connectivity between SMN-DAN is increased. (p < 0.05, FDR correction) (Fig. 4A). In state 2, the between-network connections between SMN-VN, VN-AN differed between PACG group and HC group (PACG < HC, p < 0.05, FDR correction) (Fig. 4B).

### Correlation results

**Figure 6:**
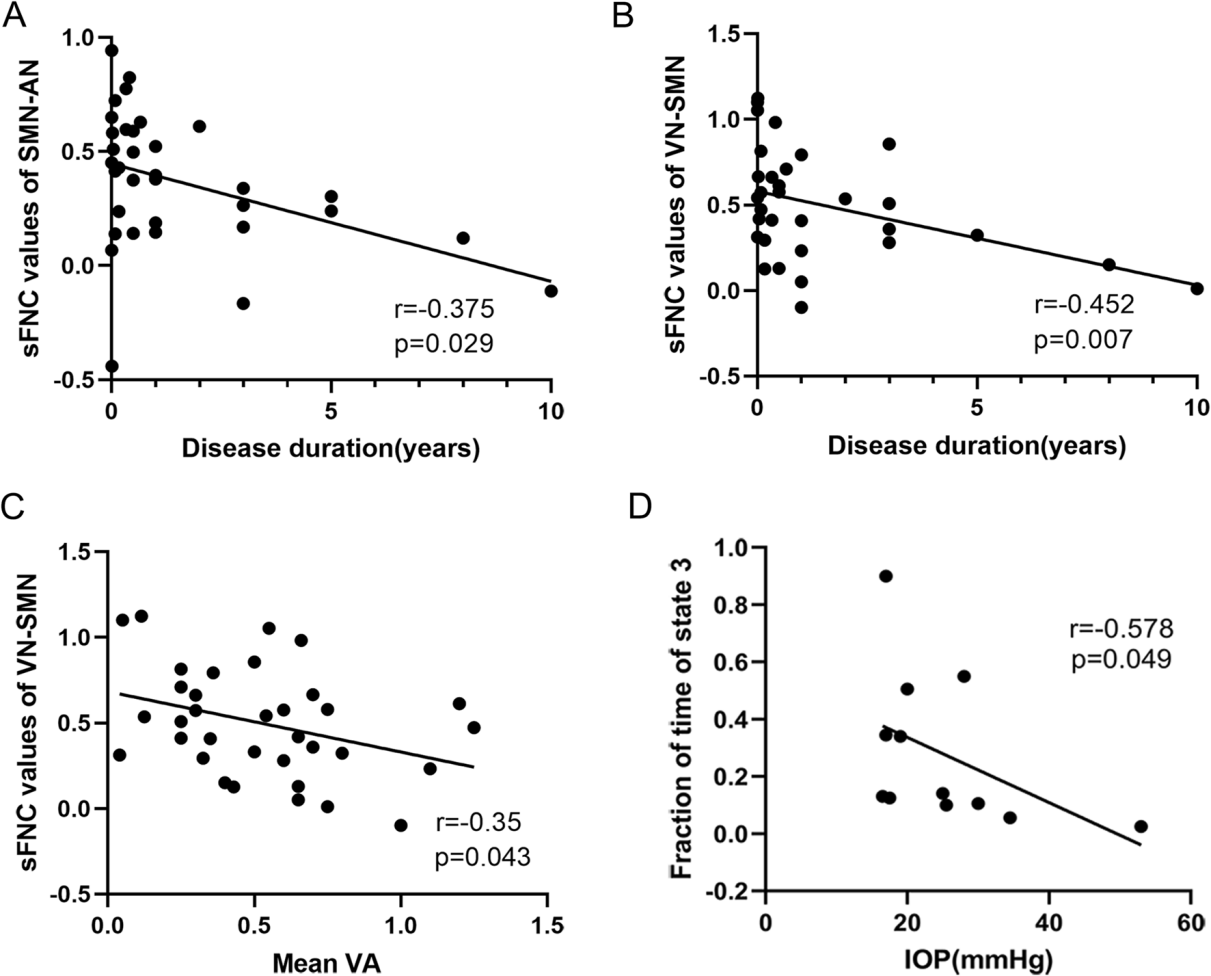
The result of correlation analysis. (**A**–**C**) Correlations between sFNC values and clinical characteristics in PACG patients. (**D**) Correlations of the PACG group between clinical characteristics and fraction of time in state 3.

The correlation between FC attributes and clinical ophthalmic parameters in the PACG group was further analyzed (Table 3 and Fig. 6). We found that the sFNC between SAM-AN was negatively correlated with the duration of disease r = −0.375, = 0.029), and the sFNC between VN-SAM was negatively correlated with the duration of disease (r = −0.452, = 0.007) and mean VA (r = −0.35, = 0.043). Additionally, the fraction of time in state 3 was negatively associated with IOP (r = −0.578, p = 0.049).

**Table 3:** Correlation analysis between PACG and HC.

FC attributes	Clinical characteristic	R values	p values
sFNC			
SMN-AN	Disease duration	− 0.375	0.029
VN-SMN	Disease duration	− 0.452	0.007
VN-SMN	Mean VA	− 0.35	0.043
dFNC			
Fraction of time of state3	IOP	− 0.578	0.049

## Discussion

The present study combined sFNC and dFNC analyses to investigate the whole brain features of PACG with a focus on the FNC states as well as the temporal properties. Our results revealed the sFNC and dFNC features and altered dynamic temporal properties, moreover, the alternations are associated with the ophthalmic parameters and duration of the disease in PACG group.

In our study, the sFNC analysis identified abnormal interactions among the Sensorimotor network (SMN), attention network (AN), and visual network (VN) in PACG patients, partially corroborating previous neuroimaging findings in this patient group^32,33^. The crucial pathological feature in PACG patients is the apoptosis of retinal ganglion cells, which unquestionably results in alterations to the visual network. Structurally, patients with glaucoma exhibit white matter tract damage far beyond visual pathway localization^34^. Functionally, PACG patients show visual dysfunction and vision-related cortex dysfunction^35,36^. These studies suggest that PACG patients have abnormalities in their visual networks, both structurally and functionally.

This study found decreased sFNC between SMN-VN. SMN play a pivotal role in the regulation of eye movement and contribute to the encoding of oculomotor activity^37^. Furthermore, prior investigations have demonstrated a significant association between the SMN and spontaneous brain activity linked to the primary visual cortex^38^, and the close connection between the SMN and the primary visual cortex is of paramount importance in the processing of spatial visual information. Similarly, a study of neovascular glaucoma observed the decreased network degree centrality in visual and sensorimotor brain regions^39^. Hence, we propose that after a decline in visual function, the SMN is affected by reduced visual signal input, suggesting potential challenges in spatial visual information processing among PACG patients. Correlation analysis showed that sFNC values between SMN-VN were negatively correlated with the duration of disease and average visual acuity. It is suggested that with the progression of the disease, the visual information processing function of PACG patients will gradually decline, and then affect the SMN.

The human brain is a complex functional connectome that segregate and integrate brain information^40^. Functional segregation entails distinct brain regions overseeing separate and relatively independent functions, while functional integration involves the collaborative processing of information across diverse brain regions, impacting perception and behavior. Gurtubay-Antolin et al.^41^ identified the potential presence of direct links between motion-sensitive regions within the visual and auditory cortices in humans. Such connections could permit an exchange of information between areas with a similar computational objective. While the primary auditory cortex is chiefly responsible for encoding auditory stimulus attributes, the secondary auditory cortex synthesizes and links this information, creating a specific perception^42^. Although hearing loss is not a major clinical symptom of PACG, previous studies have identified auditory dysfunction in PACG patients^9,43^. In addition, the cognitive and functional deterioration of the individual were tied not just to the loss of auditory and visual capabilities, but also to dual deficiency^44^. This study found decreased sFNC between AN and VN, AN and SMN, which indicates that patients with PACG may have a decline in cognitive function and disruption of sensorimotor information integration in vision and hearing. Besides, the sFNC values of AN-SMN were negatively correlated with the duration of disease, suggesting that the integration function of brain networks may be significantly diminished in advanced PACG patients.

In comparing dFNC differences within RSNs between groups, we found the results were highly similar to those of sFNC, with subtle differences between the different states. In state 1, the dFNC values between SMN-VN, SMN-AN, AN-VN were reduced. And in state 2, the dFNC values between SMN-VN, AN-VN were reduced. The results showed that the dynamic changes of FNC in PACG patients over time was not obvious, indicating the stability of FNC in PACG patients. In addition to the observed low connectivity changes akin to sFNC, we identified a specific increase in connectivity between SMN and DAN in state 1. DAN is considered to be the “aperture” of the brain and is primarily responsible for goal-directed attention control in visual attention tasks. A task-based fMRI study revealed that DAN activity correlates with reliance on visual cues^45^. In other words, the more dependent on the timing and content of the visual cues, the stronger DAN's activity. Thus, the connectivity between SMN-DAN is enhanced in PACG patients, possibly to compensate for the reduced input of visual information.

We identified four stable and repetitive dFNC states in all subjects and compared differences in three temporal features in each state. State 1 has the highest proportion in all time Windows, with the longest mean dwell time and generally lower connectivity. This indicated that PACG patients were predominantly located in state 1 and characterized the baseline activity of neurons in the resting brain. State 2 demonstrates a notably interconnected state overall, but no significant difference was found in the three temporal characteristics. This may be due to the low proportion of state 2 in all time Windows. The fraction of time in state 3 was reduced in patients with PACG compared with HCs, suggesting that PACG patients were more likely to transition from state 3 to other states. This reduction implies abnormal information transfer within or between functional networks, ultimately suggesting abnormal local segregation and global integration of functional brain networks. And this finding has also been observed in many neurodegenerative and psychiatric disorders^46–48^. The connectivity pattern of state 4 is characterized by a modular connection, primarily evidenced by a clear positive connection between DMN and VN, while a distinct negative connection exists between DMN and AN. This suggests that in state 4, patients with PACG primarily exhibit a compensatory state.

Correlation analysis showed that the fraction of time in state 3 of PACG patients was negatively correlated with the IOP. The results are consistent with previous studies^49^, but it needs to be interpreted with caution because less than half of patients with PACG showed evidence of state 3 in the rs-fMRI data. Short-term variation in IOP represents the nyctohemeral pattern and has been related to continuous progression of glaucoma^50^. Therefore, we believe that the decreased fraction of time in state 3 may indicate disease progression in patients with PACG. In addition, IOP is an essential risk factor for glaucoma management. The effect of surgical treatment and prognosis deterioration in PACG patients correlates with elevated IOP. Hence, the fraction of time in state 3 of PACG patients may be a brain imaging marker to judge the prognosis of PACG patients. Certainly, these findings pertain solely to alterations of functional network, and structural networks still need to be further discussed in the future.

Several limitations in this study warrant consideration. Firstly, the brain functional networks were based on a limited number of network models, excluding others like the cerebellum network, which may be crucial in understanding the pathophysiology of optic nerve impairment in PACG. Future studies should include a more comprehensive range of network interaction models. Secondly, the impact of emotional factors was not assessed, as patients with PACG often experience varying degrees of depression and other emotional changes. Future research should incorporate detailed psychological evaluations. Lastly, the lack of longitudinal research means the specific temporal relationship between brain activity alterations and disease stage remains unclear, and the study had a small sample size. Future studies should consider increasing the sample size and grouping PACG patients based on the duration of their disease.

## Conclusions

This study explored for the static and dynamic FNC patterns in patients with PACG. Compared with HCs, PACG patients exhibit abnormalities in both sFNC and dFNC. The high degree of overlap between static and dynamic results suggests the stability of functional connectivity networks in PACG patients. Besides, the temporal properties (fraction of time) of PACG were altered, and correlated with clinical ophthalmic parameters. These results suggest that PACG patients may have dyfunction in network separation and integration, which provide a new perspective to understand the neuropathological mechanisms of optic nerve damage in PACG patients.

## Data Availability

The data that support the findings of this study are available from the First Affiliated Hospital of Nanchang University but restrictions apply to the availability of these data, which were used under license for the current study, and so are not publicly available. Data are however available from the corresponding author upon reasonable request and with permission of the First Affiliated Hospital of Nanchang University.
